# Autoantibody levels are associated with acute kidney injury, anemia and post-discharge morbidity and mortality in Ugandan children with severe malaria

**DOI:** 10.1038/s41598-019-51426-z

**Published:** 2019-10-17

**Authors:** Juan Rivera-Correa, Andrea L. Conroy, Robert O. Opoka, Anthony Batte, Ruth Namazzi, Benson Ouma, Paul Bangirana, Richard Idro, Andrew L. Schwaderer, Chandy C. John, Ana Rodriguez

**Affiliations:** 1 Department of Microbiology, New York University School of Medicine, New York, NY 10010 USA; 2 Department of Pediatrics, Indiana University School of Medicine, Indianapolis, IN 46202 USA; 3 Department of Pediatrics and Child Health, Makerere University, Kampala, Uganda; 4 Child Health and Development Centre, Makerere University, Kampala, Uganda; 5 Department of Medical Microbiology, College of Health Sciences, Makerere University, Kampala, Uganda; 6 Department of Psychiatry, Makerere University, Kampala, Uganda; 7 Centre of Tropical Medicine and Global Health, University of Oxford, Oxford, UK; 8 Department of Pediatrics, University of Minnesota, Minneapolis, USA

**Keywords:** Infection, Acute kidney injury, Malaria

## Abstract

Autoantibodies targeting host antigens contribute to autoimmune disorders, frequently occur during and after infections and have been proposed to contribute to malaria-induced anemia. We measured anti-phosphatidylserine (PS) and anti-DNA antibody levels in 382 Ugandan children prospectively recruited in a study of severe malaria (SM). High antibody levels were defined as antibody levels greater than the mean plus 3 standard deviations of community children (CC). We observed increases in median levels of anti-PS and anti-DNA antibodies in children with SM compared to CC (p < 0.0001 for both). Children with severe malarial anemia were more likely to have high anti-PS antibodies than children with cerebral malaria (16.4% vs. 7.4%), p = 0.02. Increases in anti-PS and anti-DNA antibodies were associated with decreased hemoglobin (p < 0.05). A one-unit increase in anti-DNA antibodies was associated with a 2.99 (95% CI, 1.68, 5.31) increase odds of acute kidney injury (AKI) (p < 0.0001). Elevated anti-PS and anti-DNA antibodies were associated with post-discharge mortality (p = 0.031 and p = 0.042, respectively). Children with high anti-PS antibodies were more likely to have multiple hospital readmissions compared to children with normal anti-PS antibody levels (p < 0.05). SM is associated with increased autoantibodies against PS and DNA. Autoantibodies were associated with anemia, AKI, post-discharge mortality, and hospital readmission.

## Introduction

Malaria is an intravascular parasitic disease caused by infection with *Plasmodium*. *Plasmodium falciparum* is the most prevalent malaria species in Africa. In 2017 there were 219 million cases and 435,000 global malaria deaths with 61% of deaths occurring in children less than five years of age^1^. Two common complications of severe malaria (SM) are severe malarial anemia (SMA) and cerebral malaria (CM). SMA is life-threatening and accounts for over half of childhood malaria deaths in Africa, and occurs through multiple mechanisms, including: direct destruction of infected RBCs, clearance of infected and uninfected RBCs, and insufficient bone marrow production (reviewed in^2^). Death is preventable in SMA if children receive an appropriate transfusion^3,4^. CM is characterized by an unrousable coma with no other identifiable cause, and typically has a mortality rate of 18–21%^5^. Reported mortality in African children with SM was 10% in the AQUAMAT study and ranged from 4–15% across 11 sites in 9 different African countries^4^.

Elevations in autoantibodies targeting self antigens, such as DNA, are well recognized in autoimmune disorders like systemic lupus erythematosus (SLE) and are used for diagnosis, prognosis, and understanding disease pathogenesis^6,7^. Autoantibodies have been associated with complications in a number of infections^8,9^, and autoimmunity during and after infection is frequently reported in diseases associated with systemic inflammatory responses— including malaria^10,11^. While autoantibodies in malaria were initially attributed to non-specific polyclonal immune activation^12^, more recent studies demonstrate direct parasite induced secretion of autoantibodies in the host^13^.

Malaria shares clinical features with autoimmune disorders, including autoimmune anemia. Using a mouse model, it was observed that anti-phopsphatidylserine (PS) antibodies promote anemia during malaria through the binding to PS exposed in uninfected erythrocytes which facilitates their clearance^14^. It was also observed that levels of anti-PS antibodies are correlated with malarial anemia in patients^14,15^. Although autoantibodies have been reported in experimental models of malaria and patient populations^16–18^, few studies have investigated the relationship between autoantibodies and SM complications. Acute kidney injury (AKI) is a common complication in pediatric SM associated with significant morbidity and mortality^4,19,20^, but the pathogenesis is not well understood.

Given reports of elevated autoantibodies in SM and clinical similarities between malaria and autoimmune disorders, we sought to evaluate the relationship between anti-PS and anti-DNA antibodies in children with SM. We hypothesized children with SM would have elevated autoantibodies associated with complications on admission including anemia and AKI. In this prospective cohort study, we measured admission anti-PS and anti-DNA autoantibodies in Ugandan children with SM and evaluated whether autoantibodies were associated with disease severity and outcomes over two years follow-up.

## Methods

### Study population

The study was performed at Mulago Hospital, Kampala, Uganda between 2008 and 2013. All eligible children between 18 months and 12 years of age were enrolled. CM was defined as: (1) coma (Blantyre Coma Score (≤2); (2) *P*. *falciparum* on blood smear; and (3) no other known cause of coma (e.g., meningitis, a prolonged postictal state or hypoglycemia-associated coma reversed by glucose infusion). SMA was defined as presence of *P*. *falciparum* on blood smear in children with a hemoglobin ≤5 g/dL. Additional exclusion criteria for children with SMA included: (1) impaired consciousness on physical exam; (2) other clinical evidence of central nervous system (CNS) disease; or (3) >1 seizure prior to admission. Children with SM were managed according to Ugandan Ministry of Health treatment guidelines at the time of the study as described^21^.

Community children (CC) were recruited from the nuclear family, extended family, or household compound area of children with SM who were between the ages of 18 months and 12 years, and within 1 year of age of the household participant with CM or SMA. We excluded children who lived further than 50 km from Mulago and children for whom the primary caregiver reported: (1) chronic illness requiring medical care, (2) history of developmental delay, (3) history of coma, head trauma, or cerebral palsy, or (4) history of hospitalization for malnutrition. Additional exclusion criteria for CC included: (1) illness requiring medical care within the previous 4 weeks or active illness; or (2) major medical or neurological abnormalities on screening physical exam.

Children were followed for two years and were asked to return to the hospital for neurodevelopmental follow up assessment at 6, 12, and 24 months. Children were also asked to return to hospital for any illness requiring treatment, and received free care and transportation reimbursement for all hospital visits.

### Assessment of acute kidney injury

The bedside Schwartz equation was used to estimate kidney function where the estimated glomerular filtration rate (eGFR) = (k*height)/Cr, where k = 0.413). Acute kidney injury (AKI) was defined using the Kidney Disease: Improving Global Outcomes (KDIGO) guidelines^22^ using on admission creatinine levels and predicted baseline creatinine from the CC^23^. A 1.5-fold increase in creatinine from baseline was defined as AKI. AKI was further staged as follows: stage 1, 1.5–1.9x increase from baseline; stage 2, 2.0–2.9 increase in creatinine from baseline; stage 3, ≥3.0x increase in creatinine from baseline or an eGFR <35 mL/min/1.73 m^2^. Data on urine output or proteinuria are not available.

### Antibody testing

Antibodies against malaria-specific antigens were measured using a multiplex magnetic bead assay as previously described^24^, and data expressed as arbitrary units (AU). The recombinant *P*. *falciparum* antigens used for testing were apical membrane antigen-1 (AMA-1, full length ectodomain FVO strain; provided by David E. Lanar, Walter Reed Army Institute for Research); glutamate rich protein (GLURP, conserved non-repeat N-terminal region, amino acids 25–514, R0; provided by Michael Theisen, Statens Seruminstitut, Copenhagen, Denmark); merozoite surface protein-1 (MSP-142 FVO strain; provided by David Narum, National Institutes of Health); and circumsporozoite protein (CSP, (NANP)_5_ repeat peptide; Sigma Genosys). Magnetic beads were coupled to four *P*. *falciparum* antigens: AMA-1 (bead region, 44; coating concentration, 2 μg), CSP (bead region, 64; coating concentration, 10 μg), GLURP (bead region, 26; coating concentration, 0.5 μg) and MSP-1 (bead region, 36; coating concentration, 1 μg). Serum samples were diluted 1:100 in serum dilution buffer, and combined 1:1 with beads to produce a final dilution of 1:200. A minimum of 50 beads/region were counted per sample and median fluorescence intensity was used as the output value. The assay used a pool of plasma samples from highly exposed Ugandan individuals (n = 30) and North American donor samples without a history of malaria exposure (n = 9).

To measure autoimmune antibodies, Costar 3750 96-well ELISA plates were coated with PS (Sigma) at 20 μg/ml in 200-proof Molecular Biology ethanol or calf thymus DNA (Sigma) at 10 μg/ml in PBS. Plates were allowed to evaporate during 16 h at 4 °C. Plates were washed 3 times with PBS 0.05% Tween-20 and then blocked for 1 h with PBS 3% BSA. Plasma from patients or control was diluted at 1:100 in blocking buffer and incubated for 2 h at 37 °C. Plates were washed again 3 times and incubated with a polyclonal sheep anti-human IgG-HRP diluted 1:2000 (GE Healthcare) for 1 h at 37 °C. Plates were washed 3 more times and developed using TMB substrate (BD Biosciences). The reaction was stopped using Stop buffer (Biolegend) and absorbance read at 450 nm. The mean OD at 450 nm from replicate wells was compared with reference serum from a Colombian *P*. *vivax* patient previously identified as high responder for anti-PS IgG antibodies to calculate relative units (RU). To determine cutoff values for anti-PS and anti-DNA antibodies, antibody reactivity was determined using the CC and a value above the group mean plus 3 standard deviations was considered to have high antibody levels.

### Statistical analysis

Data were analyzed using STATA v14.0 (StataCorp., 2015) and GraphPad Prism v7. Unadjusted comparisons for continuous measures using the Wilcoxon rank sum test or categorical measures using Pearson’s Chi square or Fisher’s exact test as appropriate. Logistic or linear regression was used to evaluate the association between autoantibody levels and SM complications. Logistic regression was used to assess the relationship between antibody levels and disease outcome adjusting for child age. A non-parametric test for trend was used to evaluate the association between autoantibodies across stages of AKI. Spearman’s rho was used to evaluate correlations between autoantibody levels and continuous laboratory or biochemical measures of malaria disease severity. Holm’s correction was used to adjust for multiple comparisons.

### Ethics statement

Written informed consent was obtained from parents or guardians of all study participants. Ethical approval was granted by the Institutional Review Boards for human studies at Makerere University School of Medicine and the University of Minnesota. All research was performed in accordance with relevant guidelines and regulations.

### Meetings where information has previously been presented

American Society of Microbiology, 2018 meeting, Atlanta, Georgia. Presenter: Juan Rivera-Correa (poster).

## Results

**Figure 1:**
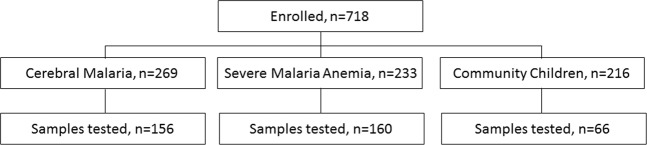
Flow chart of study participants.

In total, 382 children with samples available for autoantibody testing were included in the study (Fig. 1). The demographic characteristics of the population are presented in Table 1. Differences in enrollment characteristics of children included versus those not included are compared in Supplementary Table 1. Overall, children with SM were younger, and had lower weight-for-height and weight-for-age z scores than CC (Table 1). Children with malaria had laboratory values consistent with a diagnosis of SM, including: lower hemoglobin and platelet counts, higher white blood cell counts, and evidence of renal dysfunction (higher creatinine and BUN) and hemolysis (elevated LDH and total bilirubin) (Table 1).

**Table 1:** Demographic characteristics of the population at enrollment.

	CC (n = 66)	SMA (n = 160)	CM (n = 156)	CC vs. Severe Malaria P-value	CM vs. SMA P-value
**Demographics**					
Age, years	3.9 (2.8, 6.0)	2.7 (2.0, 4.1)	3.5 (2.6, 4.9)	0.001*	0.0004*
Female sex, (%) No.	34 (51.5)	59 (36.9)	66 (42.3)	0.073	0.323
Weight-for-age z score	−0.9 (−1.4, −0.1)	−1.5 (−2.3, −0.7)	−1.2 (−1.9, −0.5)	0.0004*	0.045
Height-for-age z score	−1.0 (−1.7, −0.2)	−1.0 (−1.8, −0.2)	−0.4 (−1.3, 0.7)	0.621	0.006
Weight-for-height z score	−0.3 (−0.8, 0.2)	−0.9 (−1.8, −0.2)	−1.4 (−2.0, −0.5)	<0.00001*	0.689
Socioeconomic status score	9 (7, 11)	9 (7, 11)	9 (8, 12)	0.350	0.664
Sickle cell disease, No. (%)	0 (0.0)	17 (10.6)	0 (0.0)	0.052	<0.0001*
HIV positive, No. (%)	1 (1.5)	4 (2.5)	3 (2.0)	1.000	1.000
**Laboratory Characteristics**					
Hemoglobin	11.8 (10.8, 12.6)	3.7 (3.0, 4.5)	6.6 (5.1, 8.5)	<0.00001*	<0.00001*
WBC	8.8 (6.5, 10.4)	11.7 (8.9, 17.4)	9.4 (6.7, 13.2)	<0.00001*	<0.00001*
Platelet count	364 (260, 429)	148 (93, 242)	61 (34, 107)	<0.00001*	<0.00001*
Glucose	—	6.4 (4.7, 8.2)	6.7 (5.3, 8.9)	—	0.143
Lactate	—	5.0 (3.0, 7.8)	3.8 (2.0, 6.8)	—	0.009
Creatinine	0.31 (0.26, 0.36)	0.35 (0.27, 0.45)	0.40 (0.30, 0.50)	0.0001*	0.012
BUN	7 (6, 10)	13 (9, 20)	17 (12, 25)	<0.00001*	0.002*
Lactate dehydrogenase	261 (234, 304)	765 (607, 1053)	829 (639, 1130)	<0.00001*	0.168
Total bilirubin	0.2 (0.1, 0.3)	1.3 (0.7, 2.0)	1.7 (0.9, 3.0)	<0.00001*	0.006
Parasite density	0 (0, 0)	34760 (10040, 133720)	41090 (13320, 294620)	<0.00001*	0.023
Plasma HRP2	4.8 (4.8, 59.4)	867 (310, 2649)	2658 (1009, 5398)	<0.00001*	<0.00001*

Data presented as median (IQR) unless otherwise indicated.

Abbreviations: community children (CC), severe malarial anemia (SMA), cerebral malaria (CM), human immunodeficiency virus (HIV), hemoglobin, g/dL; white blood cell (WBC), ×10^3^/μL; platelet count, ×10^3^/μL; glucose, mmol/L; lactate, mmol/L; creatinine, mg/dL; blood urea nitrogen (BUN), mg/dL; lactate dehydrogenase, U/L; total bilirubin, mg/dL; parasite density, parasites/uL; plasma histidine rich protein 2 (HRP2), ng/mL.

*Significant following Holm’s adjustment for multiple comparisons (n = 36).

Within the SM group, children with CM had higher median hemoglobin levels, lower white blood cell and platelet counts, and higher parasite biomass compared to children with SMA (adjusted p < 0.05) (Table 1). We further characterized the differences between children with CM and SMA based on the number of WHO severe malaria criteria present (Supplementary Table 2). Children with CM were more likely to be prostrate, have repeated convulsions, and hyperparasitemia (adjusted p < 0.05) compared to children with SMA (Supplementary Table 2). On the other hand, children with SMA were more likely to have jaundice (adjusted p < 0.05). Overall, children with CM had more severe malaria criteria present than children with SMA (Supplementary Table 2).

### Autoantibody levels are elevated in children with severe malaria compared to CC

**Figure 2:**
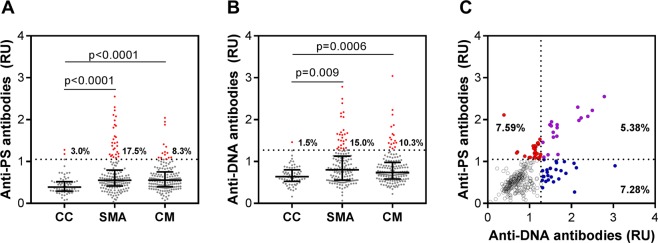
Anti-PS and anti-DNA antibody levels are elevated in children with severe malaria compared to community controls. (**A**,**B**) Scatter plot with interquartile range of anti-PS and anti-DNA antibodies in Ugandan children with severe malaria (severe malarial anemia (SMA), n = 160; cerebral malaria (CM), n = 156) or community children (CC, n = 66) by ELISA expressed as relative units (RU). Samples were considered positive for autoantibodies if the relative units (RU) > the mean plus 3 times the standard deviation of the CC. The cut-off is indicated by the horizontal line, and positive samples are indicated in red. The group prevalence of positive autoantibodies is indicated to the right of the distribution. Differences continuous autoantibody levels between groups analyzed using the non-parametric Wilcoxon rank sum test. (**C**) Scatter plot showing the correlation between anti-PS and anti-DNA antibodies in children with severe malaria (rho, 0.558, p < 0.0001) showing the percentage of children considered positive for each antibody: blue (anti-DNA antibodies), red (anti-PS antibodies), and purple (anti-PS and anti-DNA antibodies positive).

We compared anti-PS and anti-DNA antibody levels at enrollment in children with SM and CC. Children with CM and SMA had higher median levels of circulating anti-PS and anti-DNA autoantibodies compared to CC (adjusted p < 0.05, Fig. 2A,B). Further, children with SM were more likely to have high autoantibodies than CC (anti-PS antibodies, 13.0% in SM vs. 3.0% in CC vs. p = 0.02; anti-DNA antibodies, 12.7% in SM vs. 1.5% in CC vs. p = 0.008). Children with SM had a 4.77 (95% CI: 1.12, 20.24, p = 0.034) and 9.42 (95% CI: 1.27, 69.79, p = 0.028) fold increase in odds of a high anti-PS and anti-DNA test compared to CC. The relationship between anti-PS and anti-DNA antibodies are shown in Fig. 2C. A total of 64 children with SM had high autoantibodies: 7.59% for anti-PS antibodies alone, 5.38% for both anti-PS and anti-DNA antibodies, and 7.28% for anti-DNA antibodies alone.

### Relationship between autoantibody levels and severe malaria complications

To determine whether autoantibody levels were related to specific malaria complications and laboratory measures of disease severity on admission, we compared anti-PS and anti-DNA antibody levels in WHO-defined SM complications (Table 2). Following correction for multiple comparisons, a one unit increase in relative units of anti-PS or anti-DNA antibodies was associated with a −1.04 g/dL (95% CI, −1.63, −0.44) and a −0.95 g/dL (95% CI, −1.52, −0.38) decrease in hemoglobin, respectively (adjusted p < 0.05). These results indicate a strong correlation between both autoantibodies and anemia. Additionally, a one unit increase in anti-DNA antibodies was associated with a 2.99 (95% CI, 1.68, 5.31) increase in the odds of AKI, a relationship not seen with anti-PS antibodies (Table 2). We repeated the analysis based on whether a child had high autoantibody levels and obtained similar results (Supplementary Table 3).

**Table 2:** Association between a one-unit increase in anti-PS and anti-DNA antibodies and SM criteria.

Clinical Complications	Anti-PS Antibodies		Anti-DNA Antibodies	
	Odds Ratio (95% CI)	P value	Odds Ratio (95% CI)	P value
Prostration	0.93 (0.52, 1.67)	0.813	0.75 (0.43, 1.30)	0.306
Coma	0.59 (0.34, 1.04)	0.067	0.70 (0.41, 1.18)	0.180
Repeated convulsions	0.49 (0.25, 0.96)	0.038	0.79 (0.44, 1.42)	0.434
Deep breathing	1.68 (0.71, 3.97)	0.234	1.20 (0.49, 2.96)	0.692
Acute kidney injury	1.51 (0.85, 2.69)	0.160	**2**.**99 (1**.**68**, **5**.**31)**	<**0**.0001*
Jaundice	1.32 (0.74, 2.36)	0.341	1.90 (1.04, 3.44)	0.034
Shock^a^	—	—	—	—
Abnormal bleeding	0.04 (0.0006, 3.16)	0.151	0.70 (0.07, 7.12)	0.763
**Laboratory Measures**	**Beta (95% CI)**	**P value**	**Beta (95% CI)**	**P value**
Hemoglobin, g/dL	**−1**.**04 (−1**.**63**, **−0**.**44)**	**0**.001*	**−0**.**95 (−1**.**52**, **−0**.**38)**	**0**.001*
Lactate, mmol/L	0.88 (−0.12, 1.87)	0.083	1.31 (0.32, 2.30)	0.010
Glucose, mmol/L	−0.55 (−1.38, 0.28)	0.192	−0.73 (−1.56, 0.11)	0.087
Parasite Density/uL	−24767 (41267)	0.549	−34350 (39542)	0.386

Logistic regression for clinical complications and linear regression for laboratory measures.

^a^Model unstable because event (n = 1).

*Significant following Holm’s correction for multiple comparisons (n = 22).

We further explored correlations between autoantibody levels and measures of disease severity in Table 3. Both Anti-PS and anti-DNA autoantibodies were positively correlated with creatinine, blood urea nitrogen (BUN), and lactate dehydrogenase (LDH) (adjusted p < 0.05). There was no correlation between anti-PS or anti-DNA antibody levels and parasite density (anti-DNA antibodies, rho, 0.042, p = 0.463; anti-PS antibodies, rho, −0.036, p = 0.526), and a weak correlation between parasite biomass (plasma HRP2) and anti-PS antibody levels (Table 3), suggesting the autoimmune response is not being driven by parasite burden.

**Table 3:** Correlation of anti-PS and anti-DNA antibodies with measures of malaria severity.

	Anti-PS Antibodies			Anti-DNA Antibodies		
	N	rho	P value	N	rho	P value
WBC	309	0.135	0.017	309	0.139	0.014
Platelet count	310	−0.049	0.394	310	0.027	0.640
Creatinine	**309**	**0**.**166**	**0**.003*	**309**	**0**.**269**	<**0**.0001*
BUN	**314**	**0**.**239**	<**0**.0001*	**314**	**0**.**208**	**0**.**0002**
Total bilirubin	307	0.051	0.370	307	0.028	0.626
Lactate dehydrogenase	**305**	**0**.**291**	<**0**.0001*	**305**	**0**.**225**	**0**.0001*
Plasma HRP2	316	0.147	0.009	316	0.002	0.974

Abbreviations: white blood cell (WBC), blood urea nitrogen (BUN), histidine rich protein 2 (HRP2).

*Significant following Holm’s correction for multiple comparisons (n = 14).

### Relationship between autoantibody levels and kidney function

As anti-DNA antibody levels were elevated in AKI, and anti-DNA and anti-PS autoantibodies correlated with creatinine and BUN, we further explored the association between autoantibody levels and kidney function. We observed an increase in anti-DNA (p < 0.001) and anti-PS (p = 0.028) antibodies across AKI stages using a non-parametric test for trend. There was an inverse relationship between anti-PS (rho, −0.206, p = 0.0003) and anti-DNA (rho, −0.283, p < 0.0001) antibodies and eGFR in children with SM, but not in CC (anti-PS, rho, −0.254, p = 0.050; anti-DNA, rho, 0.049, p = 0.709). There was an increase in the frequency of children with high anti-DNA antibodies across AKI stages (no AKI, 9.3%; stage 1 AKI, 16.7%; stage 2 AKI, 18.5%; stage 3 AKI, 41.7%), p = 0.005.

In a sub-set of children who had hemoglobinuria assessed (n = 260), there was an increase in median anti-DNA antibodies in children with hemoglobinuria (p = 0.017) compared to children without hemoglobinuria. There was no change in anti-PS antibody levels in children based on the presence of hemoglobinuria on admission (p = 0.294). There was no relationship between serum albumin levels and autoantibody levels (anti-DNA antibodies, rho, 0.020, p = 0.727; anti-PS antibodies, rho, −0.036, p = 0.521) and proteinuria was not assessed.

To determine whether the relationship between reduced glomerular filtration and autoantibodies was related to systemic changes in IgG metabolism, we compared levels of antibodies (IgG) to malaria-specific antigens representing the blood stage of infection: (i) apical-membrane antigen 1 (AMA-1); (ii) merozoite surface protein 1 (MSP-1), and (iii) glutamate-rich protein (GLURP); and for the liver stage: (iv) circumsporozoite protein (CSP) (Table 4). In children with SM, there were no differences in median levels of antibodies against AMA-1, MSP-1, GLURP, or CSP in children with AKI (p > 0.05 for all). In addition, there were no differences levels of malaria-specific IgG and eGFR in SM using linear regression (Table 4, p > 0.05 for all).

**Table 4:** Correlation between malaria antibodies and measures of malaria severity.

	AMA-1			MSP-1			CSP			GLURP		
	N	rho	P value	N	rho	P value	N	rho	P value	N	rho	P value
Hemoglobin	254	−0.045	0.477	254	0.156	0.013	254	−0.052	0.410	254	0.004	0.955
WBC	247	−0.007	0.910	247	−0.035	0.589	247	0.085	0.186	247	0.183	0.004
Platelet count	248	−0.098	0.124	**248**	**−0.232**	0.0002*	248	−0.074	0.248	248	−0.032	0.617
Creatinine	248	−0.082	0.195	248	−0.017	0.795	248	0.133	0.037	248	0.101	0.114
BUN	254	−0.082	0.195	254	−0.078	0.214	254	0.080	0.203	254	−0.047	0.461
Total bilirubin	**248**	**−0.250**	0.0001*	248	−0.143	0.0246	248	−0.045	0.486	248	−0.063	0.323
Lactate dehydrogenase	247	−0.132	0.038	247	−0.017	0.006	247	0.013	0.846	247	−0.100	0.118
Plasma HRP 2	254	−0.156	0.013	254	−0.040	0.432	254	−0.028	0.662	254	−0.144	0.022
eGFR	247	0.140	0.028	247	0.035	0.580	247	−0.124	0.051	247	−0.042	0.509

Abbreviations: apical membrane antigen-1 (AMA-1), merozoite surface protein-1 (MSP-1), circumsporozoite protein (CSP), glutamate rich protein (GLURP), white blood cell (WBC), blood urea nitrogen (BUN), histidine rich protein 2 (HRP2), estimated glomerular filtration rate (eGFR).

*Significant following Holm’s correction for multiple comparisons (n = 36).

Finally, we evaluated whether there were changes in malaria-specific antibodies and markers of disease severity (Table 4). In contrast to autoimmune antibodies that were positively correlated with several measures of disease severity (creatinine, BUN, LDH) (Table 3), there were no clear differences in relationships between malaria-specific antibodies and disease severity (Table 4). In general, we observed inverse relationships between antibody levels and measures of disease severity (e.g. higher bilirubin with reduced AMA-1 levels). However, many of these relationships were not significant following adjustment for multiple comparisons.

### Relationship between autoantibody levels and clinical recovery and survival

As anti-PS antibodies have previously been shown to promote anemia in a mouse model and correlate with malarial anemia in *P*. *falciparum* patients^14,15^, we evaluated if there was a relationship between high autoantibody levels and hospital readmission during the study period. Children with high anti-PS antibodies were more likely to be readmitted during the study period compared to children without high anti-PS antibodies (35.9% vs. 20.5%, p = 0.032). Further, children with high anti-PS antibodies were more likely to be readmitted to the hospital multiple times with an adjusted OR of 3.84 (95% CI: 1.44, 10.23, p = 0.007) following correction for age (Table 5). There was no relationship between anti-DNA antibodies and hospital readmission (p > 0.05).

**Table 5:** Relationship between anti-PS and anti-DNA antibodies and outcomes.

	Anti-PS Antibodies		Anti-DNA Antibodies	
	Odds Ratio (95% CI)	P value	Odds Ratio (95% CI)	P value
**Autoantibody levels**				
In-hospital mortality	0.81 (0.38, 1.73)	0.587	0.63 (0.22, 1.82)	0.393
Post-discharge mortality	4.15 (1.03, 16.81)	0.046	4.50 (0.87, 23.38)	0.073
Hospital readmission	1.40 (0.89, 2.22)	0.147	0.78 (0.43, 1.42)	0.425
Multiple readmissions	1.88 (0.88, 3.99)	0.102	1.03 (0.40, 2.68)	0.944
**High autoantibody levels**				
In-hospital mortality	0.39 (0.05, 3.01)	0.366	0.40 (0.05, 3.10)	0.382
Post-discharge mortality	3.48 (0.61, 19.73)	0.159	3.67 (0.64, 20.92)	0.143
Hospital readmission	1.95 (0.94, 4.06)	0.074	0.38 (0.13, 1.10)	0.075
Multiple readmissions	3.84 (1.44, 10.23)	0.007	0.33 (0.04, 2.50)	0.280

^a^Logistic regression model adjusting for child age.

*Significant following Holm’s correction for multiple comparisons (n = 16).

Given the relationship between autoantibody levels and disease severity, we evaluated whether autoantibodies were related to in-hospital and post-discharge all-cause two year survival (Table 5). There was no difference in autoantibody levels in children who died in-hospital, but there was an increase in median levels of anti-PS and anti-DNA antibody levels in children who died post-discharge (p = 0.03 and p = 0.04, respectively). A one unit increase in the natural log of anti-PS and anti-DNA antibody levels was associated with increased odds of two-year post-discharge mortality (odds ratio (OR) (95% CI): anti-PS, 4.15 (1.03, 16.81), p = 0.046; anti-DNA, 4.50 (0.87, 23.38), p = 0.073) following adjustment for child age. Although the cause of post-discharge mortality was not always ascertained (as a number of children died outside of health centers), all 11 children who died in follow up had severe anemia during their initial hospitalization. Two of the follow-up deaths occurred in children with CM on admission (both with severe anemia and AKI), and neither had neurologic abnormalities on discharge.

## Discussion

In the present study we evaluate the relationship between autoimmune antibodies, SM complications, and in-hospital and post-discharge mortality in a cohort of Ugandan children with SM and CC. Since autoimmune anti-PS antibodies promote malaria-induced anemia in a mouse model and are correlated with malarial anemia in adults^14,15^, we hypothesized that these antibodies may also play a role in children with malarial anemia and that other autoimmune antibodies, such as anti-DNA, may contribute to malaria pathogenesis. In this work, we demonstrate that in children with SM, anti-PS antibodies are strongly associated with anemia on admission, post-discharge mortality, and hospital readmissions, and that anti-DNA antibodies are associated with AKI. Together, the study findings suggest that in children with SM, anti-PS antibodies may play a role in the development of severe anemia and post-discharge morbidity and mortality, and that immune mediated pathways may contribute to malaria-related AKI.

Previous studies have shown that anti-PS autoantibodies contribute to anemia in experimental murine models of malaria by binding to uninfected RBC and promoting their premature clearance from the circulation^14^. Analysis of plasma samples from adults with *P*. *falciparum* infections have documented an association between anti-PS antibodies andmalarial anemia^14,15^. This study extends these findings by showing anti-PS and anti-DNA autoantibodies are inversely associated with hemoglobin in children with SM during the acute phase of illness. Further, we show that children positive for anti-PS antibodies on admission are at risk for multiple hospital readmissions during follow-up. These data are consistent with previous reports describing an association of anti-RBC autoantibodies and anemia in *P*. *vivax*^25,26^, although in these studies only antibodies recognizing proteins, not lipids or nucleic acids (such as PS and DNA), were analyzed.

In mouse models of malaria, infected, but also uninfected RBC, expose PS in their surface^14,27^, which is recognized by anti-PS antibodies, contributing to anemia^14^. Our knowledge of this mechanism in human patients is more limited, but an inverse correlation of anti-PS antibodies and hemoglobin levels was described in malarial anemia^14,15^. Since *in vitro* treatment of RBC with artesunate increases PS translocation to the RBC surface^28^, it is possible anti-PS antibodies binding to PS on the surface of RBC may be an additional mechanism contributing to anemia following artesunate treatment^29^. Additional studies are needed to determine: (i) how levels of anti-PS autoantibodies change over infection; (ii) whether anti-PS antibodies may contribute to recurrent anemia in children with repeated malaria infections; and (iii) whether anti-PS autoantibodies accelerate the removal of RBC following parenteral treatment with artesunate.

We observed a correlation between lactate dehydrogenase (LDH) and autoantibody levels, suggesting RBC lysis may be mediated by autoantibodies. Alternatively, it is possible hemolysis enhances autoantibody production through immune activation and exposure to self-antigens. Additional studies are needed to delineate whether RBC hemolysis is mediated by complement fixation on RBC-bound anti-PS antibodies, or whether hemolysis triggers the generation of autoantibodies.

Anti-DNA antibodies also correlated with anemia in children with SM. This association may be explained by the observations that DNA is abundant in the circulation of malaria patients^30^ and that cell-free DNA binds to uninfected red blood cells^31^. RBC-bound DNA could be targeted by infection-induced anti-DNA antibodies promoting their clearance and enhancing anemia. Cell-free DNA in malaria patients declines upon resolution^32^, which could explain the lack of correlation of anti-DNA antibodies with hospital readmission.

In this study anti-DNA antibodies were strongly associated with the presence and severity of AKI. Previous studies in children with SM suggest the etiology of AKI at presentation is largely related to reduced renal perfusion^19^ as a result of hypovolemia and impaired microvascular perfusion from parasitized RBC cytoadhesion to the endothelium. We observed an inverse relationship between eGFR and anti-DNA and anti-PS antibodies during SM, raising questions on whether reduced autoantibody clearance in AKI leads to increases in circulating autoantibodies. The MHC class 1-like Fc receptor (FcRn) is one receptor important in maintaining serum levels of albumin and IgG, contributing to the long half-life of IgG. However, podocytes and proximal tubule epithelium in the kidney express FcRn that have been shown to promote IgG clearance from the glomerular basement membrane^33^. Additionally, other receptors involved in antibody removal such as complement receptor 1 (CR1/CD35) are expressed on podocytes^34^ and down regulated in other cell types during malaria^35^. While it is possible that reduced GFR may lead to altered renal handling of IgG and affect levels of circulating autoantibodies, we did not observe any relationship with total IgG against malaria antigens AMA-1, MSP-1, CSP, or GLURP and any measures of renal function (AKI, creatinine, BUN, or eGFR). These data suggest changes in IgG metabolism are not responsible for the increase in autoantibodies.

Anti-DNA antibodies are associated with glomerulonephritis and AKI in SLE through a variety of mechanisms including: (a) DNA immune complex (IC) entrapment in glomerular basement membranes, (b) non-DNA antigen crossreactivity, (c) binding to apoptoic cellular debris in the glomerulus and acting as “a planted antigen” and/or (d) anti-DNA binding to kidney cells including podocytes, endothelial cells and/or proximal tubule cells leading to renal injury^36^. *P*. *malariae* can lead to a nephrotic syndrome associated with a membrano-proliferative glomerulonephritis with deposits of IgM, IgG and C3 in mesangial and subendothelial tissue^37^. However, the pathogenesis of *P*. *falciparum* appears to be different and the role of auto-antibodies and IC in kidney pathogenesis during *P*. *falciparum* remains unclear. Even if it is well-documented that there are increases in circulating DNA and anti-DNA that form IC in the circulation^32^, evidence of IC deposition in kidneys of patients with *P*. *falciparum* is inconsistent^38–40^. The relationship between anti-DNA autoantibodies and glomerulonephritis has not been systematically investigated using immunofluorescent labeling of immunoglobulins and complement to detect and characterize glomerulonephritis in renal biopsies of surviving patients. Together, these results indicate that an immune mediated pathway, possibly an immune-mediated glomerulonephritis, similar to what is observed with SLE nephritis, could contribute to *P*. *falciparum* malaria-associated AKI^41^. We further speculate that anti-DNA autoantibodies may lead to proximal tubular cell injury and acute tubular necrosis in the absence of glomerulonephritis.

This study has several strengths. We were able to measure autoantibodies in a well characterized population of Ugandan children with SM and compare autoantibodies with malaria-specific antibodies. The finding of a strong association between anti-DNA autoantibodies and AKI (defined using established KDIGO criteria and using CC to define baseline creatinine), that was not present for anti-PS antibodies or malaria-specific antibodies, suggests a mechanistic link between anti-DNA antibodies and AKI. Further, the prospective design enabled us to evaluate the relationship between autoantibodies, post-discharge mortality, and risk of subsequent hospitalization.

This study has limitations. We included children with two manifestations of severe malaria and therefore cannot comment on the generalizability of these findings to other manifestations of severe or uncomplicated malaria. As we did not collect longitudinal samples, we cannot draw conclusions on how autoantibody levels change following treatment and whether there are clinical consequences associated with post-treatment autoantibody levels. Apart from the assessment of hemoglobin in the urine, no additional urinalysis was conducted and urine was not stored. Quantification of blood and protein in the urine and renal biopsy findings will be needed to better characterize AKI in SM.

In summary, we report elevations in anti-DNA and anti-PS autoantibodies in SM. Consistent with another study in malarial anemia^14,15^, we show a relationship between autoantibodies and anemia, this time in children with acute severe malaria in an endemic area. We also show that children testing positive for anti-PS antibodies were more likely to be readmitted to the hospital and readmitted multiple times compared to children negative for anti-PS antibodies. We also report a relationship between anti-DNA antibodies and the presence and severity of AKI. Additional studies are needed to evaluate the impact of malaria treatment on autoantibody levels, and to assess the relationship between post-treatment autoantibodies and long-term complications of severe malaria including recurrent hospitalizations for severe anemia, death, and chronic kidney disease.

## Supplementary information

Supplementary Tables
