# Supplementary material for: Autoantibody levels are associated with acute kidney injury, anemia and post-discharge morbidity and mortality in Ugandan children with severe malaria
Source: Sci Rep. 2019 Oct 17;9:14940. doi: 10.1038/s41598-019-51426-z (PMC6797715; doi:10.1038/s41598-019-51426-z)
Supplement: Supplementary file 1 — Supplementary Tables [file 41598_2019_51426_MOESM1_ESM.docx]

**Supplementary Information for:** Autoantibody levels are associated with acute kidney injury, anemia and post-discharge morbidity and mortality in Ugandan children with severe malaria.

**Author List:** Juan Rivera-Correa^1^*, Andrea L. Conroy^2^*, Robert O. Opoka^3^, Anthony Batte^4^, Juan Rivera-Correa^1^*, Andrea L. Conroy^2^*, Robert O. Opoka^3^, Anthony Batte^4^, Ruth Namazzi^3^, Benson Ouma^5^, Paul Bangirana^6^, Richard Idro^3,7^, Andrew L. Schwaderer^2^, Chandy C. John^2,8^ and Ana Rodriguez^1^

^1^Department of Microbiology, New York University School of Medicine, New Y, NY 10016, USA; ^2^Department of Pediatrics, Indiana University School of Medicine, Indianapolis, IN, 46202, USA; ^3^Department of Pediatrics and Child Health, Makerere University, Kampala, Uganda;

^4^Child Health and Development Centre, Makerere University, Kampala, Uganda; ^5^Department of Medical Microbiology, College of Health Sciences, Makerere University, Kampala, Uganda;

^6^Department of Psychiatry, Makerere University, Kampala, Uganda; ^7^Centre of Tropical Medicine and Global Health, University of Oxford, Oxford, UK. ^8^Department of Pediatrics, University of Minnesota, Minneapolis, USA.

* Authors contributed equally

**Supplementary Table 1. Comparison of children included in the study**

|  | Severe Malaria | | | Community Children | | |
| --- | --- | --- | --- | --- | --- | --- |
|  | **Included** | **Not Included** | **P value** | **Included** | **Not Included** | **P value** |
| Demographics |  |  |  |  |  |  |
| Age, years | 3.2 (2.2, 4.4) | 3.4 (2.3, 4.8) | 0.521 | 3.9 (2.8, 6.0) | 3.5 (2.6, 4.5) | 0.514 |
| Female sex, (%) No. | 125 (39.6) | 78 (41.9) | 0.600 | 34 (51.5) | 78 (52.0) | 0.948 |
| Weight-for-age z score | -1.3 (-2.1, -0.6) | -1.4 (-2.0, -0.5) | 0.517 | -0.9 (-1.4, -0.1) | -0.9 (-1.6, -0.2) | 0.243 |
| Height-for-age z score | -0.9 (-1.7, 0.01) | -1.1 (-2.0, -0.2) | 0.067 | -1.0 (-1.7, -0.2) | -1.2 (-1.9, -0.4) | 0.699 |
| Weight-for-height z score | -1.0 (-1.8, -0.3) | -0.9 (-1.6, 0.2) | 0.040 | -0.3 (-0.8, 0.2) | -0.4 (-1.0, 0.4) | 0.080 |
| Socioeconomic status score | 9 (7, 11) | 8 (7, 10) | 0.099 | 9 (7, 11) | 9 (8, 12) | 0.638 |
| Sickle cell disease, No. (%) | 17 (5.4) | 6 (3.2) | 0.265 | 0 (0) | 0 (0) | ---- |
| HIV positive, No. (%) | 7 (2.3) | 4 (2.3) | 0.971 | 1 (1.5) | 2 (1.3) | 0.916 |
| Laboratory Characteristics |  |  |  |  |  |  |
| Hemoglobin | 4.7 (3.6, 6.6) | 5.0 (4.0, 7.4) | 0.014 | 11.8 (10.8, 12.6) | 11.9 (11.0, 12.5) | 0.232 |
| WBC | 10.7 (7.6, 15.2) | 10.1 (7.4, 15.4) | 0.385 | 8.8 (6.5, 10.4) | 8.9 (7.4, 10.9) | 0.226 |
| Platelet count | 100 (51, 191) | 94 (43, 162) | 0.125 | 364 (260, 429) | 385 (296, 466) | 0.226 |
| Glucose | 6.6 (5.0, 8.7) | 6.4 (4.5, 8.0) | 0.335 | ---- | ---- | ---- |
| Lactate | 4.5 (2.3, 7.2) | 4.0 (2.8, 7.7) | 0.892 | ---- | ---- | ---- |
| Creatinine | 0.37 (0.29, 0.47) | 0.42 (0.31, 0.55) | 0.004 | 0.31 (0.26, 0.36) | 0.29 (0.24, 0.35) | 0.160 |
| BUN | 15 (10, 22) | 16 (10, 22) | 0.717 | 7 (6, 10) | 7 (6, 9) | 0.904 |
| Lactate dehydrogenase | 789 (621, 1097) | 772 (639, 1056) | 0.775 | 261 (234, 304) | 261 (227, 318) | 0.750 |
| Total bilirubin | 1.4 (0.8, 2.4) | 1.4 (0.8, 2.5) | 0.941 | 0.2 (0.1, 0.3) | 0.1 (0.1, 0.2) | 0.006 |
| Parasite density | 38460 (11340, 187300) | 48940 (9660, 210100) | 0.374 | 0 (0, 0) | 0 (0, 0) | 0.121 |
| Plasma HRP-2 | 1667 (527, 4001) | 2261 (720, 4709) | 0.073 | 4.8 (4.8, 59.4) | 4.8 (4.8, 119.4) | 0.554 |

Abbreviations: human immunodeficiency virus (HIV), hemoglobin, g/dL; white blood cell (WBC), x10^3^/μL; platelet count, x10^3^/μL; glucose, mmol/L; lactate, mmol/L; creatinine, mg/dL; blood urea nitrogen (BUN), mg/dL; lactate dehydrogenase, U/L; total bilirubin, mg/dL; parasite density, parasites/uL; plasma histidine rich protein 2 (HRP-2), ng/mL.

Data presented as median (IQR) unless otherwise indicated.

*Significant following Holm’s adjustment for multiple comparisons (n=36)

**Supplementary Table 2. World Health Organization severe malaria criteria by group**

|  | All SM (n=316) | SMA (n=160) | CM (n=156 ) | P value |
| --- | --- | --- | --- | --- |
| Prostration | 224 (70.9) | 68 (42.5) | 156 (100.0) | <0.0001* |
| Coma | 156 (49.4) | 0 (0.0) | 156 (100.0) | <0.0001* |
| Repeated convulsions | 97 (30.7) | 1 (0.6) | 96 (61.5) | <0.0001* |
| Deep breathing | 25 (7.9) | 8 (5.0) | 17 (10.9) | 0.052 |
| Acute kidney injury | 93 (30.2) | 37 (23.9) | 56 (36.6) | 0.015 |
| Jaundice | 200 (63.3) | 119 (74.4) | 81 (51.9) | <0.0001* |
| Shock | 1 (0.3) | 0 (0.0) | 1 (0.6) | 0.494 |
| Severe anemia | 195 (61.7) | 160 (100.0) | 35 (22.4) | <0.0001* |
| Lactic acidosis | 128 (44.0) | 72 (49.0) | 56 (38.9) | 0.083 |
| Hypoglycemia | 15 (4.9) | 7 (4.6) | 8 (5.2) | 0.811 |
| Abnormal bleeding | 5 (1.6) | 1 (0.6) | 4 (2.6) | 0.210 |
| Hyperparasitemia | 34 (10.8) | 8 (5.0) | 26 (16.8) | 0.001* |
| Number of WHO SM criteria | 4 (3, 5) | 3 (2, 4) | 4 (3, 6) | <0.0001* |

Abbreviations: severe malaria (SM), severe malarial anemia (SMA), cerebral malaria (CM), World Health Organization (WHO)

* Significant following Holm’s adjustment for multiple comparisons (n=13)

**Supplementary Table 3. Association between positive autoantibodies and severe malaria criteria**

|  | Positive anti-PS antibodies | | Positive anti-DNA antibodies | |
| --- | --- | --- | --- | --- |
| Clinical Complications | **Odds Ratio (95% CI)** | **P value** | **Odds Ratio (95% CI)** | **P value** |
| Prostration | 0.99 (0.48, 2.04) | 0.981 | 0.45 (0.23, 0.88) | 0.020 |
| Coma | 0.43 (0.21, 0.86) | 0.018 | 0.65 (0.33, 1.27) | 0.207 |
| Repeated convulsions | 0.42 (0.18, 0.99) | 0.048 | 0.72 (0.34, 1.55) | 0.405 |
| Deep breathing | 2.94 (1.14, 7.55) | 0.025 | 0.94 (0.27, 3.28) | 0.918 |
| Acute kidney injury | 1.18 (0.58, 2.42) | 0.648 | 2.50 (1.37, 4.95) | 0.008 |
| Jaundice | 1.29 (0.64, 2.60) | 0.477 | 1.41 (0.69, 2.89) | 0.348 |
| Shock^a^ | ---- | ---- | ---- | ---- |
| Abnormal bleeding^a^ | ---- | ---- | ---- | ---- |
| Laboratory Measures | **Beta (95% CI)** | **P value** | **Beta (95% CI)** | **P value** |
| Hemoglobin | -0.91 (-1.64, -0.17) | 0.016 | -0.91 (-1.65, -0.17) | 0.017 |
| Lactate | 0.60 (-0.61, 1.81) | 0.329 | 1.64 (0.43, 2.85) | 0.008 |
| Glucose | -0.04 (-1.05, 0.97) | 0.934 | -0.29 (-1.32, 0.74) | 0.580 |
| Parasite Density | -48066 (-146667, 50535) | 0.338 | -45164 (-145913, 55585) | 0.378 |

Logistic regression for clinical complications and linear regression for continuous laboratory measures. Hemoglobin, g/dL; lactate, mmol/L; glucose, mmol/L; parasite density, parasites/uL.

^a^Model failure to converge due to limited events
